# Partial impairment of insulin receptor expression mimics fasting to prevent diet-induced fatty liver disease

**DOI:** 10.1038/s41467-020-15623-z

**Published:** 2020-04-29

**Authors:** Troy L. Merry, Chris P. Hedges, Stewart W. Masson, Beate Laube, Doris Pöhlmann, Stephan Wueest, Michael E. Walsh, Myrtha Arnold, Wolfgang Langhans, Daniel Konrad, Kim Zarse, Michael Ristow

**Affiliations:** 1 Energy Metabolism Laboratory, Institute of Translational Medicine, Department of Health Sciences and Technology, Swiss Federal Institute of Technology (ETH), Zürich, Switzerland; 2 Discipline of Nutrition, School of Medical Sciences, The University of Auckland, Auckland, New Zealand; 3 Maurice Wilkins Centre for Molecular Biodiscovery, The University of Auckland, Auckland, New Zealand; 4 Division of Pediatric Endocrinology and Diabetology and Children’s Research Centre, University Children’s Hospital, Zurich, Switzerland; 5 Physiology and Behavior Laboratory, Institute of Food and Nutrition, Department of Health Sciences and Technology, Swiss Federal Institute of Technology (ETH), Zürich, Switzerland

**Keywords:** Biochemistry, Cell biology, Molecular biology, Physiology, Endocrinology

## Abstract

Excessive insulin signaling through the insulin receptor (IR) may play a role in the pathogenesis of diet-induced metabolic disease, including obesity and type 2 diabetes. Here we investigate whether heterozygous impairment of insulin receptor (IR) expression limited to peripheral, i.e. non-CNS, tissues of adult mice impacts the development of high-fat diet-induced metabolic deterioration. While exhibiting some features of insulin resistance, PerIRKO^+/−^ mice display a hepatic energy deficit accompanied by induction of energy-sensing AMPK, mitochondrial biogenesis, PPARα, unexpectedly leading to protection from, and reversal of hepatic lipid accumulation (steatosis hepatis, NAFLD). Consistently, and unlike in control mice, the PPARα activator fenofibrate fails to further affect hepatic lipid accumulation in PerIRKO^+/−^ mice. Taken together, and opposing previously established diabetogenic features of insulin resistance, incomplete impairment of insulin signaling may mimic central aspects of calorie restriction to limit hepatic lipid accumulation during conditions of metabolic stress.

## Abstract (web summary)

Hyper-insulinemia associated with excess calorie intake may cause metabolic dysfunction. Here the authors report that mice with partially reduced insulin receptor expression in peripheral tissues are protected from and experience reversal of fatty liver disease.

## Introduction

Obesity is associated with increased risk of developing various metabolic-related diseases such as type 2 diabetes, cardiovascular disease, cancer, and non-alcoholic fatty liver disease (NAFLD)^1,2^. The molecular causes of obesity-driven metabolic disease are not fully understood but are often coupled with the development of insulin resistance in peripheral tissue, a pathophysiological manifestation driving metabolic disease^3–5^. Insulin acts via the insulin receptor (IR)/phosphatidylinositol 3-kinase (PI3K)/AKT2 signaling pathway to facilitate glucose transport from the blood into skeletal muscle and adipose cells for use in ATP production or storage as glycogen and triglycerides (TGs), respectively. In the liver, insulin primarily acts to suppresses hepatic glucose production and stimulate lipogenesis and glycogen synthesis^3,6^. The net effect of this coordinated response is the lowering of blood glucose levels, and if this is not achieved then pancreatic β-cells compensate by increasing insulin release resulting in hyperinsulinemia, a key characteristic of insulin resistance.

Hyperinsulinemia is fundamentally viewed as a compensatory mechanism to correct insulin resistance. Interestingly, however, studies that have attenuated high-calorie diet-induced hyperinsulinemia in mice report reduced fat mass, attenuation of NAFLD, improved insulin sensitivity, and extended lifespan^7–10^. Indeed, hyperinsulinemia often precedes the development of marked insulin resistance and fat mass gain^11–13^, and insulin infusions in rodents and humans can result in insulin resistance^14,15^, suggesting that excessive insulin signaling resulting from hyperinsulinemia may have a causative role in the development of diet-induced obesity and associated metabolic dysfunction. This is consistent with the hypothesis that hyper-insulin signaling can desensitize aspects of the insulin signaling cascade below the level of the IR^16,17^, and that impairment of insulin/IGF1 signaling leads to metabolic adaptions that extend life and health-span of *Caenorhabditis elegans*, *Drosophila melanogaster*, and several mammalian models^18–21^.

Hepatic insulin resistance is one of the earliest pathologies to develop in response to metabolic overload; however, it does not affect all insulin-signaling pathways equally. Although insulin’s ability to suppress glycogenolysis/gluconeogenesis is reduced, it continues to promote hepatic lipogenesis^22,23^. This selective insulin resistance is problematic because hyperinsulinemia will stimulate hepatic lipid storage without lowering blood glucose levels, contributing to the development of hepatic steatosis that can progress to the more serious non-alcoholic steatohepatitis. The latter reflects hepatic lipid accumulation (steatosis) combined with overt inflammation causing hepatocyte death, fibrosis, and impaired hepatic function^2^. In the current study, we questioned whether hyperinsulinemia-driven excessive insulin signaling in peripheral tissue contributes to the development of diet-mediated insulin resistance, and/or NAFLD. We have previously shown that partial peripheral tissue IR expression is sufficient for optimal insulin signaling^24^ and therefore investigated whether reducing expression of the IR in the peripheral tissue of mice fed a diet that induces obesity could protect from metabolic dysfunction. We report that partial disruption of the IR in peripheral tissues of adult mice protects from high-fat diet-induced NAFLD, and suggest that the activation of AMPK and PPARα contributes to the altered hepatic lipid metabolism.

## Results

### Partial IR disruption has limited effect on body composition

**Fig. 1:**
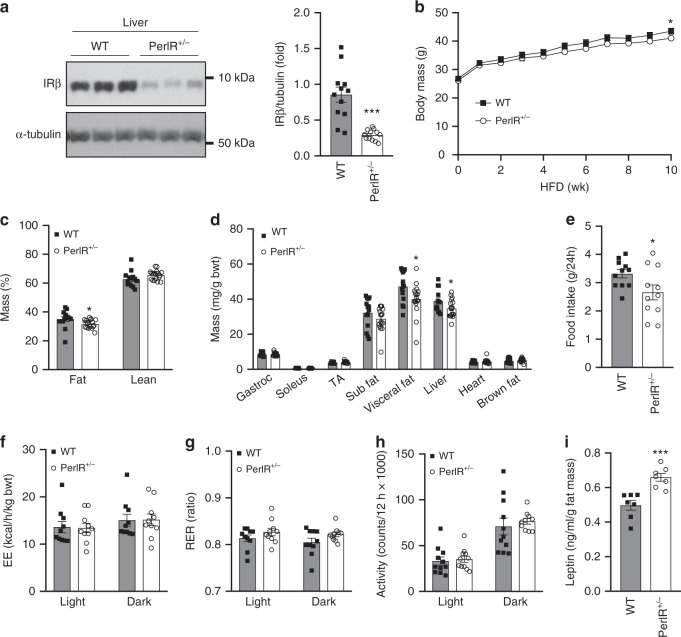
The effect of partial peripheral tissue IR disruption on body composition and energy expenditure in adult mice fed a high-fat diet. Ten days following tamoxifen (TX) treatment, male mice were fed a high-fat diet (HFD) and liver insulin receptor (IR) β expression **a**, body mass **b**, body composition **c**, **d**, food intake **e**, energy expenditure (EE) **f**, respiratory exchange ratio (RER) **g** and activity levels **h** were determined, as well as plasma leptin **i**. Results are shown as means ± SE, with *n*’s represented as individual data point’s in figures. For body weight *n* = 13 for WT and 17 for PerIRKO^+/−^. Significance was determined using two-tailed student’s *t* test. *p < 0.05, ***p < 0.001 vs WT. Gastroc: gastrocnemius muscle, TA: tibialis anterior muscle, Sub: subcutaneous, visceral fat is the epididymal fat pad.

Previously we have reported that male PerIRKO^+/−^ mice fed a chow diet show a mild reduction in body fat mass 6 weeks following induction of partial IR disruption^24^. Here we sought to determine whether the reduction in fat mass was increased during conditions that promote fat mass accumulation. After 10 weeks of HFD-feeding, male PerIRKO^+/−^ mice had a 35–65% reduction in IR expression in peripheral tissues such as visceral fat, liver, and skeletal muscle (Fig. 1a and Supplementary Fig. 1a-d) compared with HFD-fed WT mice, and HFD-induced reduction in hepatic IR expression was similar in WT and PerIRKO^+/−^ mice (Fig. 1e). We have previously reported that PerIRKO^+/−^ mice have normal IR expression in central nervous system tissues (whole brain, hypothalamus, and the pituitary)^24^. Body mass of PerIRKO^+/−^ mice was lower than WT, and this was attributed to a reduction in fat mass, with no change in lean body mass (Fig. 1b–d). This reduction was to a similar extent reported in chow-fed mice, suggesting these mice were not further protected from HFD-induced gains in fat mass^24^. The modest reduction in relative fat mass is likely associated with a reduced food intake (Fig. 1e), as there was no observable difference between WT and PerIRKO^+/−^ mice in whole-body energy expenditure, substrate utilization, or activity levels (Fig. 1f–h). Leptin is an adipokine involved in the regulation of energy intake, and complete peripheral IR ablation has previously been reported to elevate plasma leptin levels and suppress food intake^25^. Consistent with this, male PerIRKO^+/−^ mice fed a HFD had substantially elevated plasma leptin levels per gram of fat mass (Fig. 1i), but no difference in absolute levels (Supplementary Fig. 2d). Male mice are the focus for the remainder of this study because female mice fed a HFD did not gain a substantial amount of fat mass nor did they become insulin resistant.

### The effect of reduced IR expression on glucose homeostasis

**Fig. 2:**
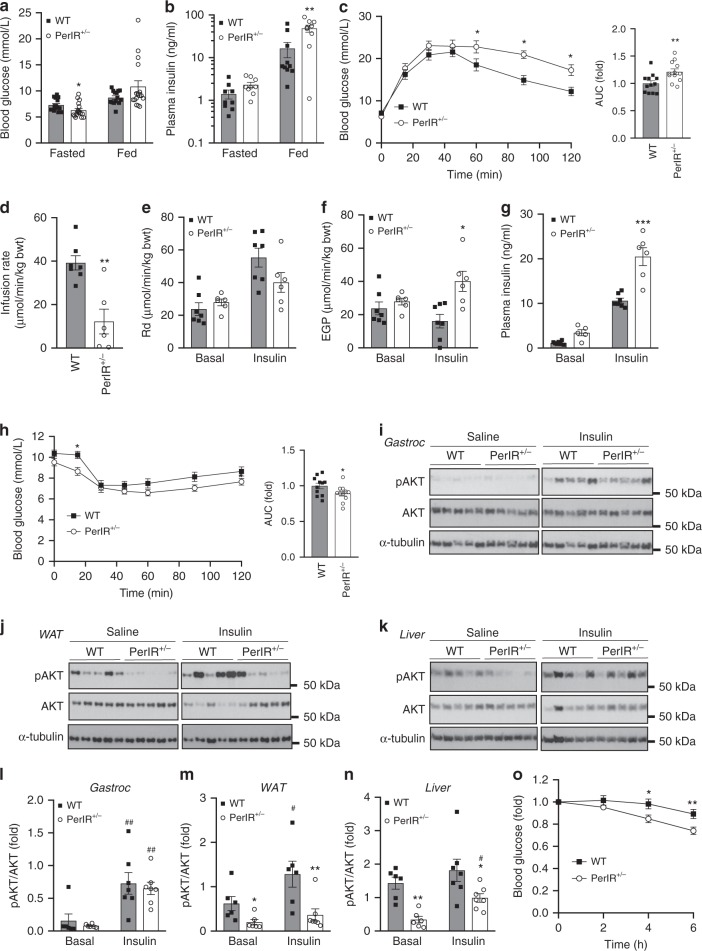
Partial peripheral tissue IR disruption alters glucose homeostasis in adult mice fed a high-fat diet. Ten days following tamoxifen (TX) treatment, male mice were fed a high-fat diet (HFD) for 10 weeks and blood was collected from fed or 6 h fasted male mice and analyzed for blood glucose or plasma insulin **a**, **b**. Glucose homeostasis and insulin sensitivity was assessed via glucose tolerance test (GTT), euglycemic–hyperinsulinemic clamp **c**–**g**, insulin tolerance test **h**, and immunoblot analysis of Ser-473 phosphorylated AKT (pAKT) and total AKT and α-Tubulin (α-Tub) in gastrocnemius (Gastroc) muscle **i**, **l**, white adipose tissue (WAT) **j**, **m** and liver **k**, **n** from WT and PerIRKO^+/−^ mice injected (intraperitoneal) with saline or insulin (0.6 mU/g). Blood glucose was monitoring during 6 h of fasting following a meal challenge **o**. Results are shown as means ± SE, with *n*’s represented as individual data point’s in figures. For fast-refeed *n* = 13 for WT and 17 for PerIRKO^+/−^. Significance was determined using two-tailed student’s *t* test or ANOVA with LSD post hoc analysis. *p < 0.05, **p < 0.01, vs WT, and ^#^*p* < 0.05, ^##^*p* < 0.01 for within genotype effect. Rd: rate of glucose disappearance, EGP: endogenous glucose production, AUC: area under curve.

As we hypothesized that hyper-insulin signaling via the IR contributes to diet-induced dysregulation of glucose homeostasis, we next determined whether partial disruption of the IR during HFD feeding alters glucose tolerance and insulin sensitivity. Despite having reduced fasted blood glucose, PerIRKO^+/−^ mice were hyperinsulinemic in the fed state (Fig. 2a, b) and less able to normalize blood glucose levels in response to a glucose bolus, suggesting glucose intolerance (Fig. 2c, Supplementary Fig. 2a). To investigate this further hyperinsulinemic–euglycemic clamps (Fig. 2d–g) and insulin tolerance tests (ITT; Fig. 2h) were performed. A lower glucose infusion rate was required to maintain blood glucose levels of PerIRKO^+/−^ (Fig. 2d) during clamp, indicating these mice had impaired insulin sensitivity. This appeared to be the result of a reduced ability for PerIRKO^+/−^ to suppress endogenous glucose production in response to insulin rather than having a lower glucose clearance (*R*_d_, Fig. 2e, f). Consistent with hepatic receptor-mediated endocytosis having a central role in clearing insulin^26^ and PerIRKO^+/−^ having impaired insulin sensitivity, at the end of the clamp PerIRKO^+/−^ plasma insulin levels were higher than that of WT mice (Fig. 2g). Surprisingly, however the ability for PerIRKO^+/−^ to lower blood glucose following an injection of insulin was not impaired (Fig. 2h, Supplementary Fig. 2b).

To assess tissue-specific insulin sensitivity the activation (phosphorylation) of AKT was determined following an insulin injection. Although insulin-induced AKT phosphorylation was similar in skeletal muscle from PerIRKO^+/−^ and WT mice (Fig. 2i, l), white adipose tissue (WAT) basal and insulin-stimulated AKT phosphorylation was substantially impaired in PerIRKO^+/−^ (Fig. 2j, m). The absolute level of hepatic AKT phosphorylation was lower in PerIRKO^+/−^ mice, yet insulin treatment induced a substantial (2.9 fold) increase in AKT phosphorylation in the livers of PerIRKO^+/−^ but not WT mice, suggesting an improved ability for insulin to induce hepatic AKT activation in PerIRKO^+/−^ (Fig. 2k, n). Consistent with overall reduced hepatic AKT activation, basal GSK3 and FOXO1 phosphorylation was lower in the liver of PerIRKO^+/−^ than WT (Supplementary Figs. 2e). While basal hepatic phosphorylation of mTORC1 targets P70SK6 and RPS6 were not affected by partial IR knockdown, PerIRKO^+/−^ tended to have greater insulin-induced RPS6 phosphorylation (Supplementary Figs. 2f).

As PerIRKO^+/−^ mice had lower fasting blood glucose and altered hepatic insulin signaling, we next determined how HFD-fed PerIRKO^+/−^ mice responded to modest increases in blood glucose by monitoring blood glucose levels following a meal. In contrast to the glucose tolerance test and hyperinsulinemic–euglycemic clamp we found that PerIRKO^+/−^ mice were better able to lower blood glucose than WT mice following a meal challenge (Fig. 2o), suggesting an improved ability to lower blood glucose in response to a physiological metabolic challenge.

**Fig. 3:**
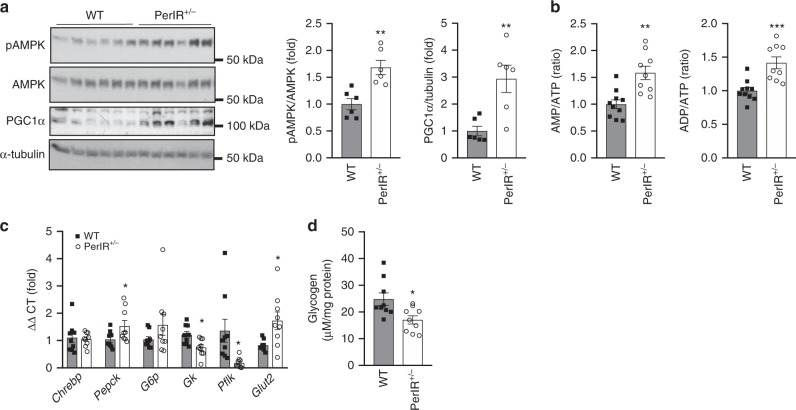
Partial peripheral tissue IR disruption induces an energy defect in the liver of adult mice fed a high-fat diet. Ten weeks following high-fat diet (HFD) feeding the livers of male WT and PerIRKO^+/−^ were collected and AMPK activation (phosphorylation), PGC1α expression **a**, ATP, ADP, AMP levels **b**, expression of glucose metabolism genes **c**, and glycogen content **d** was determined. Results are shown as means ± SE, with *n*’s represented as individual data point’s in figures. Significance was determined using two-tailed student’s *t* test; *p < 0.05, **p < 0.01, ***p < 0.001 vs WT.

As a result of enhanced insulin-induced increases in hepatic AKT phosphorylation, we next turned our attention to the liver and assessed hepatic energy status and gluconeogenic gene expression. AMP-activated protein kinase (AMPK) is activated (phosphorylated) in response to a reduction in intracellular energy status and acts to turn off energy consuming processes, while stimulating energy producing pathways^27^. PerIRKO^+/−^ mice had increased hepatic AMPK activation (phosphorylation) and PGC1α expression, which likely resulted from an increase in AMP/ATP and ADP/ATP ratio (Fig. 3a, b), indicating a hepatic energy deficit. This was not observed under chow conditions (Supplementary Fig. 3d). To understand what might be causing this energy defect, we next measured the expression of glucose regulatory genes (mRNA) in the liver. Gluconeogenic genes *Chrebp*, *Pepck,* and *G6p* were not greatly altered in PerIRKO^+/−^ mice (Fig. 3c). In contrast, the expression of the glucose metabolism genes glucokinase (*Gk*) and phosphofructokinase (*Pfkl*) was attenuated in PerIRKO^+/−^ mice, suggesting reduced glycolysis and availability of glucose as substrate (Fig. 3c). In agreement, PerIRKO^+/−^ mice had reduced hepatic glycogen (Fig. 3d) and what is likely to be a compensatory increase in gene expression of the major hepatic glucose transporter, *Glut2* (Fig. 3c). These effects were not observed under chow-fed conditions (Supplementary Fig. 3a).

### Partial IR disruption prevents and reverses fatty liver

**Fig. 4:**
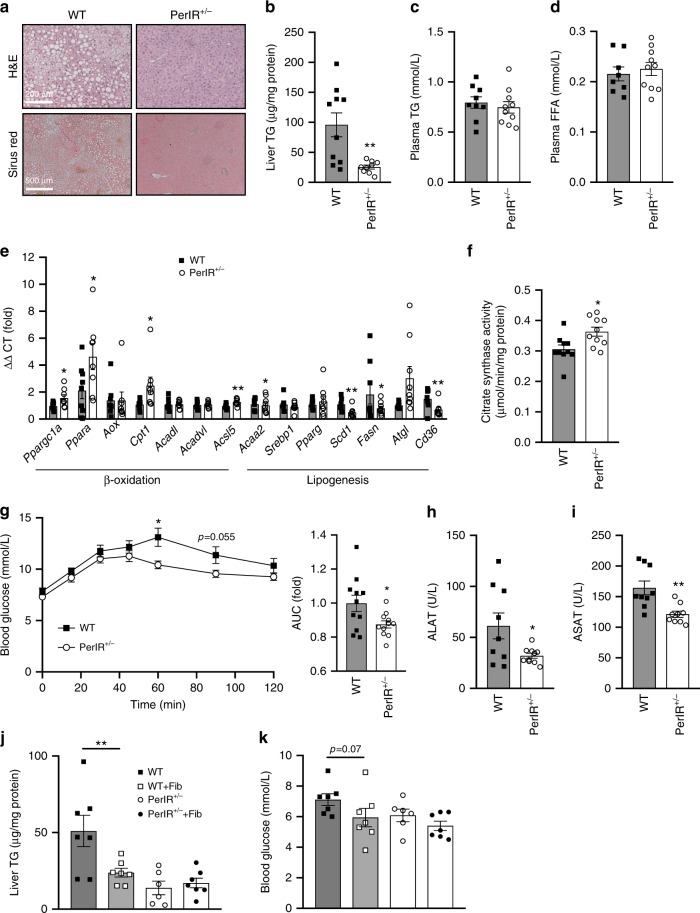
The effect of partial peripheral tissue IR disruption on liver function in adult mice fed a high-fat diet. Livers from male WT and PerIRKO^+/−^ mice fed a high-fat diet (HFD) for 10 weeks were processed for H&E, Sirius red staining, assayed for triglyceride content **a**, **b**, and plasma triglycerides (TG) and free fatty acids (FFA) were measured **c**, **d**. The expression of genes associated with lipid metabolism, and citrate synthase activity was determined **e**, **f**, a pyruvate tolerance test was performed **g** and plasma samples were analyzed for the liver damage enzymes ALAT, ASAT **h**, **i**. Ten days following tamoxifen (TX) treatment, male mice were fed a high-fat diet (HFD) for 10 weeks with or without the PPARα agonist fenofibrate (Fib) and liver TG **j** and fasted blood glucose **k** was determined. Results are shown as means ± SE, with *n*’s represented as individual data point’s in figures. Significance was determined using two-tailed student’s *t* test or ANOVA with LSD post hoc analysis; *p < 0.05 and **p < 0.01 vs WT.

As PerIRKO^+/−^ mice showed altered expression of hepatic glucose metabolism genes, we next assessed whether partial loss of the peripheral tissue IR might affect hepatic lipid metabolism. Indeed, hepatic TG accumulation was substantially reduced in PerIRKO^+/−^ mice independent of plasma TGs and free fatty acids (FFA) (Fig. 4a–d). Consistent with elevated hepatic lipid turnover and the activation of AMPK, we observed increased expression of β-oxidation genes, reduced expression of lipogenesis genes, as well as elevated mitochondrial citrate synthase activity in the livers of PerIRKO^+/−^ mice (Fig. 4e, f). This effect was not observed under normal chow diet conditions (Supplementary Fig. 3c, and^24^). We then sought to determine whether reduced hepatic steatosis in PerIRKO^+/−^ was associated with improved liver function. PerIRKO^+/−^ showed an attenuated blood glucose response to i.p injection of pyruvate, suggesting increased ability to suppress hepatic gluconeogenesis, and reduced systemic markers of liver damage (ALAT and ASAT) (Fig. 4g–i).

We next hypothesized that the energy defect in the liver of PerIRKO^+/−^ mice was activating AMPK, which was regulating hepatic lipid metabolism via the transcription factors PPARα and PGC1α. To assess the contribution of PPARα, mice were treated with the PPARα agonist fenofibrate. Fenofibrate reduced HFD-fed WT mice liver TG levels to a similar extent as PerIRKO^+/−^, and the fenofibrate treatment of PerIRKO^+/−^ mice did not result in any further reduction, or alteration in fasted blood glucose (Fig. 4j, k), potentially suggesting an overlap between these pathways.

**Fig. 5:**
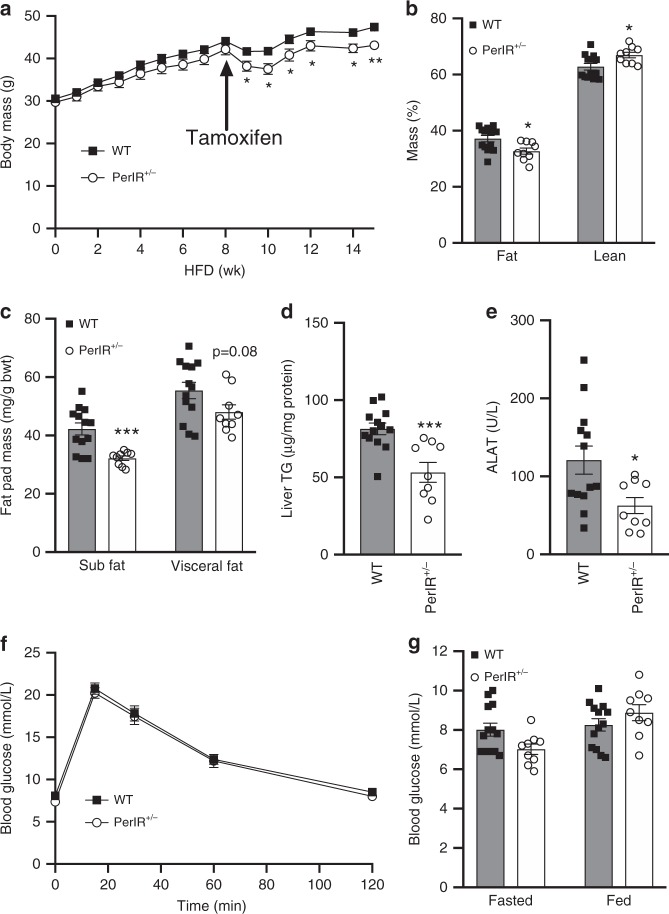
Induction of partial peripheral tissue IR disruption following high-fat diet feeding lowers hepatic lipid content without impairing glucose tolerance. Following 8 weeks of high-fat diet (HFD) feeding, mice were treated with tamoxifen and body mass **a** body composition **b**, **c**, hepatic lipid content **d** and plasma ALAT **e** were determined, and glucose homeostasis assessed via glucose tolerance test **f** and blood glucose measurement **g** 6–7 weeks later. Results are shown as means ± SE, with *n*’s represented as individual data point’s in figures. For body weight and GTT *n* = 13 for WT and 9 for PerIRKO^+/−^. Significance was determined using two-tailed student’s *t* test; *p < 0.05, **p < 0.01, and ***p < 0.001 vs WT.

To determine whether targeting the IR may have potential as a treatment for NAFLD we fed PerIRKO^+/−^ and WT mice a HFD for 8 week prior to the induction of partial peripheral tissue IR disruption (Fig. 5a). Following tamoxifen treatment, PerIRKO^+/−^ mice had slightly lower body mass than WT, which was primarily the result of a lower fat mass (Fig. 5a–c). Consistent with the hypothesis that hyperinsulinemic signaling drives NAFLD development and progression, 7 weeks following tamoxifen treatment PerIRKO^+/−^ had substantially lower hepatic lipid content and plasma ALAT (Fig. 5d, e). Surprisingly, and in contrast to when the IR was disrupted prior to HFD treatment (Fig. 2), PerIRKO^+/−^ showed similar glucose tolerance as WT mice (Fig. 5f, g).

## Discussion

Insulin is essential for regulating a large range of intracellular metabolic processes; however, chronic high levels of insulin resulting from excessive nutrient intake may also be involved in the pathogenesis of metabolic diseases like obesity, diabetes, and NAFLD. Here, we report that partially reducing the IR in the peripheral tissue of adult mice fed a high-fat diet can protect against, and reverse hepatic steatosis. In addition, we provide evidence to suggest that reducing IR expression causes an energy defect in the liver of HFD-fed mice, altering hepatic glucose and hepatic lipid metabolism.

Genetic suppression of the *INS1/INS2*^8,9^ or *GPX1*^7^ genes in mice has been reported to prevent diet-induced hyperinsulinemia and protect from fatty liver disease. This is at least partially attributed to lower insulin levels elevating energy expenditure resulting in reduced whole-body fat mass. While we report here that PerIRKO^+/−^ mice had slightly lower fat mass, this was associated with reduced food intake rather than an increase in energy expenditure. As tamoxifen does not effectively activate the Cre recombinase in the CNS of IR-floxed mice^24,25^, it would be expected that diet-induced central hyperinsulinemic signaling would be maintained and may explain why energy expenditure was not altered. However, PerIRKO^+/−^ mice had increased plasma leptin levels relative to fat mass, which is consistent with previous reports in mice with complete peripheral tissue^25^ or adipose tissue^28^ IR ablation. Moreover, leptin can act in the hypothalamus to suppress food intake^29^. It is unlikely that the resulting reduction in fat mass was great enough to prevent diet-induced hepatic lipid accumulation, suggesting an alternative mechanism of action.

Insulin resistance is characterized by the inability for insulin to act on peripheral tissue to regulate glucose homeostasis^3^, and at least early on in diet-induced development this resistance appears to develop below the level of the IR^16,17^. In the liver, insulin resistance selectively impairs insulin-induced suppression of hepatic glucose production while insulin’s ability to suppress β-oxidation and promote lipogenesis remains intact^16,30^. This has led to the suggestion that the hepatic insulin-signaling cascade branches to control hepatic glucose output and lipid metabolism, with the mammalian target of rapamycin complex 1 (mTORC1) signaling pathway remaining insulin-sensitive and regulating hepatic lipogenesis^31,32^. As this divergence in signaling occurs downstream of the IR, we expected that partial ablation would suppress mTORC1 activity. However, the mTORC1 targets P70S6K and RPS6 were not sustainably affected by partial IR ablation, potentially suggesting that the reduction of IR is acting primarily by altering liver glucose metabolism or through mTORC1-independent lipogenesis regulators.

Consistent with this, the absolute levels of insulin-induced signaling downstream of the IR (namely pAKT) were suppressed in PerIRKO^+/−^ mice; however, high levels of insulin (as in the postprandial state) were still capable of activating the insulin-signaling cascade. It is likely that this reduction in chronic physiological signaling associated with hyperinsulinemia, as well as altered hepatic glycogen storage and glycolysis pathway gene expression, contribute to the observed hepatic energy deprivation. Similar to physiological stressors that alter cellular energy status, such as fasting or exercise^33^, PerIRKO^+/−^ mice showed hepatic activation of AMPK and increased expression of PPARα, both of which are involved in lipid metabolism. Indeed, AMPK activators can attenuate diet-induced hepatic lipid accumulation by reducing the expression of similar hepatic lipogenesis genes (*Scd-1*, *Fasn*) to those downregulated by partial peripheral IR ablation^34^, and pharmacological activation of PPARα protects against the development of NAFLD^35,36^. As treatment of PerIRKO^+/−^ mice with the PPARα agonist fenofibrate did not have an additive effect on lowering blood glucose or hepatic lipid content, this may indicate that reduced insulin signaling protects from hepatic steatosis by activating PPARα and AMPK signaling to alter hepatic lipid metabolism during conditions of metabolic overload. However, hepatic lipid storage was already substantially reduced in the livers of HFD-fed PerIRKO^+/−^ mice, meaning that perhaps further lowering of hepatic lipid content with fenofibrate may not have been possible. This makes it difficult to ascertain whether partial peripheral IR ablation protection from NAFLD is entirely dependent on PPARα activation and warrants further investigation.

An alternative hypothesis to explain hepatic selective insulin resistance is that the liver actually remains insulin-sensitive during nutritional overload and liver cell-non-autonomous insulin-sensitive signaling controls hepatic glucose output. Indeed, the Birnbaum group^37–39^ has provided substantial evidence that insulin acts in the adipose tissue to reduce circulating FFA, which can subsequently suppress hepatic glucose output. Our findings may not fully support this as PerIRKO^+/−^ had similar FFA levels to that of WT. However, it does raise the possibility that given the mouse model we employed has reduced IR expression in peripheral tissues in addition to the liver^38,39^, that non-hepatic cells are contributing to the protection from NAFLD. Indeed, there is evidence of insulin signaling cross-talk between the liver and adipose tissue with complete IR ablation specifically in adipose tissue, leading to lipodystrophy coupled with severe hepatic lipid accumulation and dysregulation of gluconeogenesis^40^. Importantly, partial IR disruption was effective in lowering hepatic lipid storage even when induced after the onset of metabolic dysfunction (8 weeks following of HFD feeding), suggesting that regardless of the mechanism there is therapeutic potential in targeting hyperinsulinemic signaling clinically to treat NAFLD progression. Indeed, our observation that suppressing peripheral insulin signaling does not induce further impairments in glucose intolerance are consistent with previous observations that reducing insulin levels in obese mice induces fat mass loss preferentially from centrally located deposits without effecting glucose tolerance^10^, and indicate that adaptations associated with suppressing hyperinsulinemic signaling are not required prior to the onset of metabolic stress to prevent hepatic lipid accumulation.

Despite a reduced level of IR expression, insulin resistance and fed-hyperinsulinemia, PerIRKO^+/−^ mice had normal or reduced fed and fasted blood glucose levels and improved ability to shut off pyruvate-driven gluconeogenesis. This is consistent with young or adult-induced hepatic IR KO mice being normoglycemic^32,41^. However, complete IR expression appears to be required to regulate glucose homeostasis during severe hyperglycemia, with PerIRKO^+/−^ mice showing pronounced glucose intolerance when challenged with a high dose of glucose, but not in response to a meal. Although skeletal muscle is primarily responsible for insulin-induced glucose disposal, the major defect in PerIRKO^+/−^ insulin signaling was in WAT, suggesting that a suppression of adipose insulin-induced glucose uptake might be responsible for the observed glucose intolerance which is in agreement with skeletal muscle IR KO mice showing normal glucose homeostasis^42^. How this glucose intolerance was avoided by partially disrupting the IR following the development of obesity is not clear but suggests that targeting the IR may be more effective treatment than preventative option for metabolic-related pathologies.

Our findings indicate that the chronic upregulation of insulin signaling through the peripheral IR during diet-induced obesity contributes to the development of hepatic dysfunction, and that targeting the IR in peripheral tissues may be an effective approach to treat NAFLD. Future studies should address whether reducing IR expression specifically in the liver is sufficient to mediate the observed improvements in hepatic function or whether cross-talk between peripheral tissues is controlling hepatic lipid accumulation.

## Methods

### Murine breeding and housing conditions

Mice were maintained in a temperature-controlled facility with 12 h light–dark cycle and ad libitum access to water and a standard chow diet (S8022-S005, ssniff diets, Soest, Germany) or a diet with 45% of energy derived from lard (HFD, S9669-E006, ssniff diets, Soest, Germany) with or without 0.1% fenofibrate. All experiments and blood collections were undertaken in the dark phase. As described previously^24,25^, peripheral tissue inducible IR knockout mice were generated by crossing IR^lox/+^ (B6.129S4(FVB)-*Insr*^*tm1Khn*^/J) and CreER (B6.129-*Gt(ROSA)26Sor*^*tm1(cre/ERT2)Tyj*^/J) mice and backcrossing on C57Bl/6 N background resulting in IR^lox/+;CreER/+^, which are referred to as PerIRKO^+/−^, where Per stands for peripheral. IR^lox/+;+/+^ littermates were used as control (WT) mice. Cre expression was induced at 10 or 18 weeks of age by administration of 2 mg tamoxifen (Cayman Chemicals, Michigan, USA) or carrier via oral gavage for 5 consecutive days. WT and PerIRKO^+/−^ received the same dose of tamoxifen, and this resulted in the partial ablation of the IR in peripheral, but not central tissues of PerIRKO^+/−^ within 10 days (Supplementary Fig. 1 and^24^). All experiments were approved by the Canton of Zurich Veterinary Office, Switzerland.

### Biochemical assays

Unless stated otherwise, all other reagents were purchased from Sigma-Aldrich Chemicals (St. Louis, MO, USA). Blood glucose was determined using a handheld glucose meter (Bayer Contour XT Meter), plasma insulin by immunoassay (Meso Scale Discovery, Gaithersburg, MD), leptin by ELISA (Crystal Chem Inc., Chicago, Illinois, USA), and plasma FFA, TGs, ALAT, and ASAT by enzymatic reaction (Cobas Mira; Hoffmann-La Roche, Basel, Switzerland). Citrate synthase activity was measured in supernatant by examining the increase of 5,5-dithiobis-2-nitrobenzoate at a wavelength of 412 nm^43^. Liver TG were extracted from liver samples as previously described^7^ and quantified using a commercial enzymatic colorimetric assay (Roche Diagnostics), and glycogen was determined by the acid-hydrolysis method^44^. Hematoxylin–Eosin and Sirius Red staining was performed on paraffin sections from livers fixed in phosphate-buffered 4% formaldehyde. Images were acquired with ×10 objective on Axio Scope.A1 microscope (Oberkochen, Germany) equipped with an AxioCam MRc digital camera (Oberkochen, Germany). Hepatic ATP and AMP levels were determined by reverse phase high performance liquid chromatography (HPLC) as described before^45^. In brief, tissue samples were removed and snap-frozen with liquid nitrogen. Samples were homogenized in pre-chilled acetonitrile buffer to precipitate protein. Following the removal of protein for later quantification by chloroform-extraction, the metabolite-containing fraction was subjected to HPLC separation and detection. Metabolites were identified by spiking of samples with appropriate standards. Metabolite content was normalized to total protein content.

Immunoblotting was performed as described previously^7^. In brief, tissues were homogenized using an electrical handheld homogenizer in 10–20 volumes of ice cold RIPA lysis buffer (50 mm HEPES (pH 7.4), 1% (vol/vol) Triton X-100, 1% (vol/vol) sodium deoxycholate, 0.1% (vol/vol) SDS, 150 mm NaCl, 10% (vol/vol) glycerol, 1.5 mm MgCl_2_, 1 mm EGTA, 50 mm sodium fluoride, protein inhibitor cocktail (Roche, Basel, Switzerland), 1 mm phenylmethysulfonyl fluoride, 1 mm sodium vanadate), incubated for 20 min on ice and centrifuged at 20,000 × *g* for 20 min at 4 °C. The supernatants were resolved by sodium dodecyl sulfate–polyacrylamide gel electrophoresis and processed for immunoblotting by standard procedures. Antibody details are provided in supplementary table 1, and uncropped western blots can be found in the Source Data File.

### Metabolic and body composition measures

Insulin (ITT), glucose (GTT), and pyruvate (PTT) tolerance tests were performed in two (ITT) or 5 h (GTT and PTT) fasted mice by intraperitoneally injecting a bolus of insulin (0.6 mU/g; ITT), d-glucose (2 mg/g; GTT) or sodium pyruvate (1 mg/g; PTT) and tail blood glucose was measured at the time points indicated as described previously^7^. Meal challenge experiments involved fasting mice overnight (16 h, largely during light cycle) then allowing ad libitum access to food for 4 h before refasting and monitoring blood glucose for the following 6 h. PhenoMaster (TSE systems, Bad Homburg, Germany) open-circuit calorimetry system was used to measure oxygen consumption and ambulatory activity over 48 h (two light–dark cycles) following a 24–48 h acclimation period and body composition by nuclear magnetic resonance (Echo MRI-100 Body Composition Analyzer, Echo Medical Systems, Huston, USA).

### Glucose clamp studies

Glucose turnover rate was assessed in freely moving mice after 10 weeks of HFD during an euglycemic–hyperinsulinemic clamp as previously described^46^. In brief, mice were anesthetized with isoflurane, and a catheter (MRE 025, Braintree Scientific) was inserted into the right jugular vein and exteriorized at the back of the neck. After 7 days of recovery, only mice that had regained >95% of their preoperative weight were studied. After a fasting period of 5 h, 3-[3 H]glucose (0.1 μCi/min; PerkinElmer) was infused for 80 mins, and blood was collected from tail tip for basal turnover calculation. After basal sampling, insulin (18 mU/kg/min) was infused for 2 h. Euglycemia was maintained by periodically adjusting a variable infusion of 20% glucose with a syringe pump (Harvard Apparatus, Holliston, MA, USA). The glucose infusion rate was calculated as the mean of the steady-state infusion (60–90 mins) after 1 h of insulin infusion. A blood sample was collected from tail tip after steady-state infusion. The glucose turnover rate was calculated by dividing the rate of 3-[3 H]glucose infusion by the plasma 3-[3 H]glucose-specific activity. Hepatic glucose production was calculated by subtracting the glucose infusion rate from the glucose turnover rate.

### Real-time polymerase chain reaction

RNA was extracted using Trizol reagent (Invitrogen, Carlsbad, CA), and mRNA was reverse transcribed using the High Capacity cDNA Reverse Transcription Kit (Applied Biosystems, Foster City, CA). Quantitative real-time PCR was performed on a ViiA 7 Real-Time PCR System (Applied Biosystems, Foster City, CA) using the SYBR green select master mix (Applied Biosystems, Foster City, CA) and relative quantification achieved using the ΔΔCt method with 18 S ribosomal RNA as an internal control. Primer sequences used are listed in Supplementary Table 2.

### Statistical analyses

All data were presented as mean ± SEM. Statistical significance was using unpaired two-tailed Student’s *t* test and two-way ANOVA with fisher’s least significant difference post hoc analysis as indicated. The level of significance was set at *p* < 0.05 (SPSS version 20, IBM, Armonk, NY, USA).

### Reporting summary

Further information on research design is available in the Nature Research Reporting Summary linked to this article.

## Supplementary information

Supplementary Information

Reporting Summary

## Data Availability

The data that support the findings of this study are available from the corresponding author upon reasonable request. A Source Data file is available for this article.

## Source data

Source data
