# Author Correction: Partial impairment of insulin receptor expression mimics fasting to prevent diet-induced fatty liver disease

**DOI:** 10.1038/s41467-020-17520-x

**Published:** 2020-07-21

**Authors:** Troy L. Merry, Chris P. Hedges, Stewart W. Masson, Beate Laube, Doris Pöhlmann, Stephan Wueest, Michael E. Walsh, Myrtha Arnold, Wolfgang Langhans, Daniel Konrad, Kim Zarse, Michael Ristow

**Affiliations:** 1 Energy Metabolism Laboratory, Institute of Translational Medicine, Department of Health Sciences and Technology, Swiss Federal Institute of Technology (ETH), Zürich, Switzerland; 2 Discipline of Nutrition, School of Medical Sciences, The University of Auckland, Auckland, New Zealand; 3 Maurice Wilkins Centre for Molecular Biodiscovery, The University of Auckland, Auckland, New Zealand; 4 Division of Pediatric Endocrinology and Diabetology and Children’s Research Centre, University Children’s Hospital, Zurich, Switzerland; 5 Physiology and Behavior Laboratory, Institute of Food and Nutrition, Department of Health Sciences and Technology, Swiss Federal Institute of Technology (ETH), Zürich, Switzerland

**Keywords:** Biochemistry, Cell biology, Molecular biology, Physiology, Endocrinology

Correction to: *Nature Communications* 10.1038/s41467-020-15623-z, published online 29 April 2020.

This Article contained an error in Fig. 2i. The alpha-tubulin western blot for the saline-treated samples (left panel) was inadvertently duplicated from the alpha-tubulin western blot for the insulin-treated samples (right panel). This has now been replaced with the correct image. This error has now been corrected in both the PDF and HTML versions of the Article.

