# Supplementary material for: Partial impairment of insulin receptor expression mimics fasting to prevent diet-induced fatty liver disease
Source: Nat Commun. 2020 Apr 29;11:2080. doi: 10.1038/s41467-020-15623-z (PMC7190665; doi:10.1038/s41467-020-15623-z)
Supplement: Supplementary file 1 — Supplementary Information [file 41467_2020_15623_MOESM1_ESM.pdf]

Supplementary Table 1. Antibodies.

Protein	Supplier	Catalog number	Dilution
α-tubulin	Sigma-Aldrich	T6074	1:10,000
β-actin	Sigma-Aldrich	A2228	1:10,000
AKT phosphor-Ser473	CST	9271	1:1000
AKT	CST	9279	1:1000
AMPK phospho-Thr172	CST	2531	1:1000
AMPKα	CST	2532	1:1000
FOXO1 phosphor-S256	CST	9461	1:1000
FOXO1	CST	9462	1:1000
GSK3α/β phosphor-S21/9	CST	9331	1:1000
GSK3β	CST	9315	1:1000
IRβ	CST	3020	1:1000
PGC1α	Millipore	2213	1:500
P70S6K phosphor-T421/S424	CST	9204	1:1000
P70S6K	CST	2708	1:1000
PGC1α	Millipore	2213	1:500
RPS6 phosphor-S235/236	Abcam	ab12864	1:1000
RPS6	Abcam	ab40820	1:1000

CST= Cell signalling technologies

Supplementary Table 2. Mouse qPCR primer sequences.

Gene name	Forward primer	Reverse primer
18s	GATCCATTGGAGGGCAAGTCT	CCAAGATCCAACTACGAGCTTTTT
Acadl	CTTGCGATCAGCTCTTTCA	GGTACATGTGGGAGTACCCG
Acadvl	GTGGCTCTGCAAGGCTGTA	CGATTCCTGTCCTCCGTCTC
Acsl5	ACCCTTTTGATGACGACCTG	CTCCTTTGGGGTCACCTGTA
Acaa2	AACGAGGCTGGCTACTTCAA	CAGGGGCGTGAAGTTATGTT
Aox	TGAAGCCTGACGGCACGTATGTAA	TTGGACAGACTCTGAGCTGCACTT
Atgl	AACACCAGCATCCAGTTCAA	GGTTCAGTAGGCCATTCCTC
Cd36	GCCAAGCTATTGCGACATGA	ATCTCAATGTCCGAGACTTTTCAAC
Chrebp	CTGGGGACCTAAACAGGAGC	GAAGCCACCCTATAGCTCCC
Cpt1	GAACCCCAACATCCCCAAAC	TCCTGGCATTCTCCTGGAAT
Fasn	TTCCAAGACGAAAATGATGC	AATTGTGGGATCAGGAGAGC
G6p	TGCAAGGGAGAACTCAGCAA	GGACCAAGGAAGCCACATG
Gk	CCCTGAGTGGCTTACAGTTC	ACGGATGTGAGTGTTGAAGC
Glut2	GTCCAGAAAGCCCCAGATACC	GTGACATCCTCAGTTCCTCTTAG
Pepck	GTGTTTGTAGGAGCAGCCATGAGA	GCCAGTGGGCCAGGTATTTG
Pfkl	ACGAGGCCATCCAGCTCCGT	TGGGGCTTGGGCAGTGTCCT
Pppargc1a	AAGTGTGGAACTCTCTGGAACTG	GGGTTATCTTGGTTGGCTTTATG
Ppara	ACAAGGCCTCAGGGTACCA	GCCGAAAGAAGCCCTTACAG
Pparg	CAAGAATACCAAAGTGCGATCAA	GAGCTGGGTCTTTTCAGAATAATA
Scd1	TGGGTTGGCTGCTTGTG	GCGTGGGCAGGATGAAG
Srebp1c	AACGTCACTTCCAGCTAGAC	CCACTAAGGTGCCTACAGAGC

Supplementary figure 1

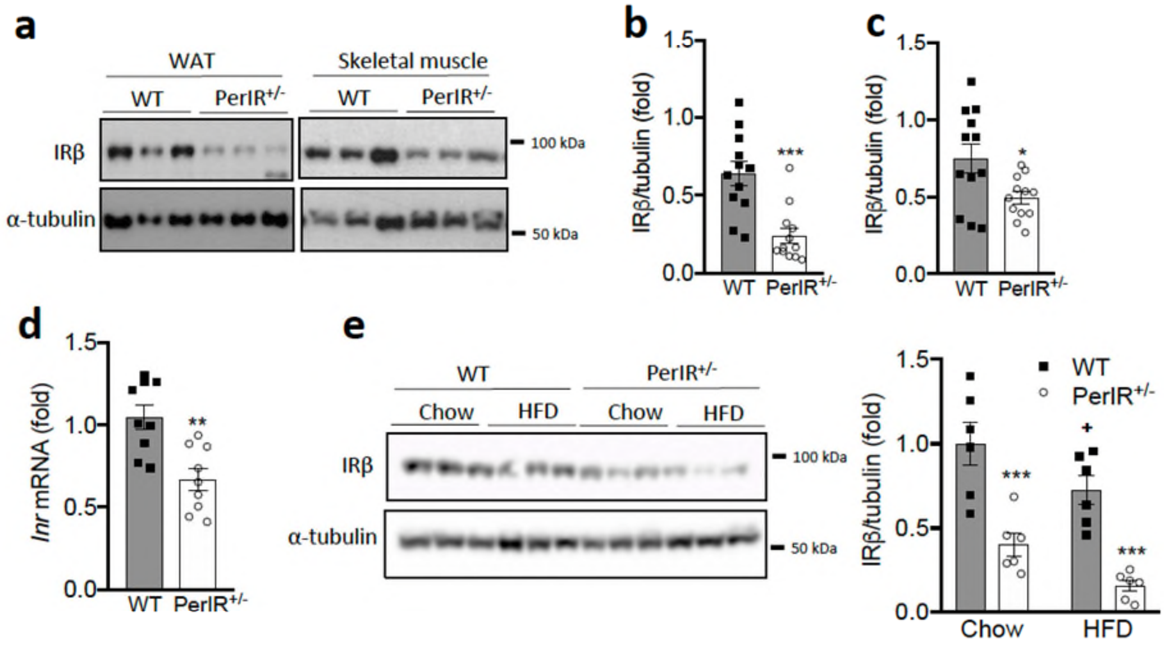

Supplementary Figure 1. Insulin receptor (IR) expression in PerIRKO$^{+/-}$ mice fed a high fat diet. Ten days following tamoxifen (TX) treatment, male mice were fed a high fat diet (HFD) for 10 weeks and white adipose tissue (WAT) and skeletal (sk.) muscle (*gastrocnemius*) insulin receptor (IR) β expression was determined **(a)**. Quantification of IRβ expression protein expression in WAT **(b)**, skeletal muscle **(c)** and hepatic mRNA **(d)** of HFD fed WT and PerIRKO$^{+/-}$ mice, and protein expression in the liver of chow and HFD WT and PerIRKO$^{+/-}$ mice **(e)**. Results are shown as means ± SE, with n's represented as individual data point's in figures. Significance was determined using two tailed student's t-test; $*p < 0.05$, $**p < 0.01$ vs WT, and $^{+}p<0.05$ vs chow diet of the same genotype.

Supplementary figure 2

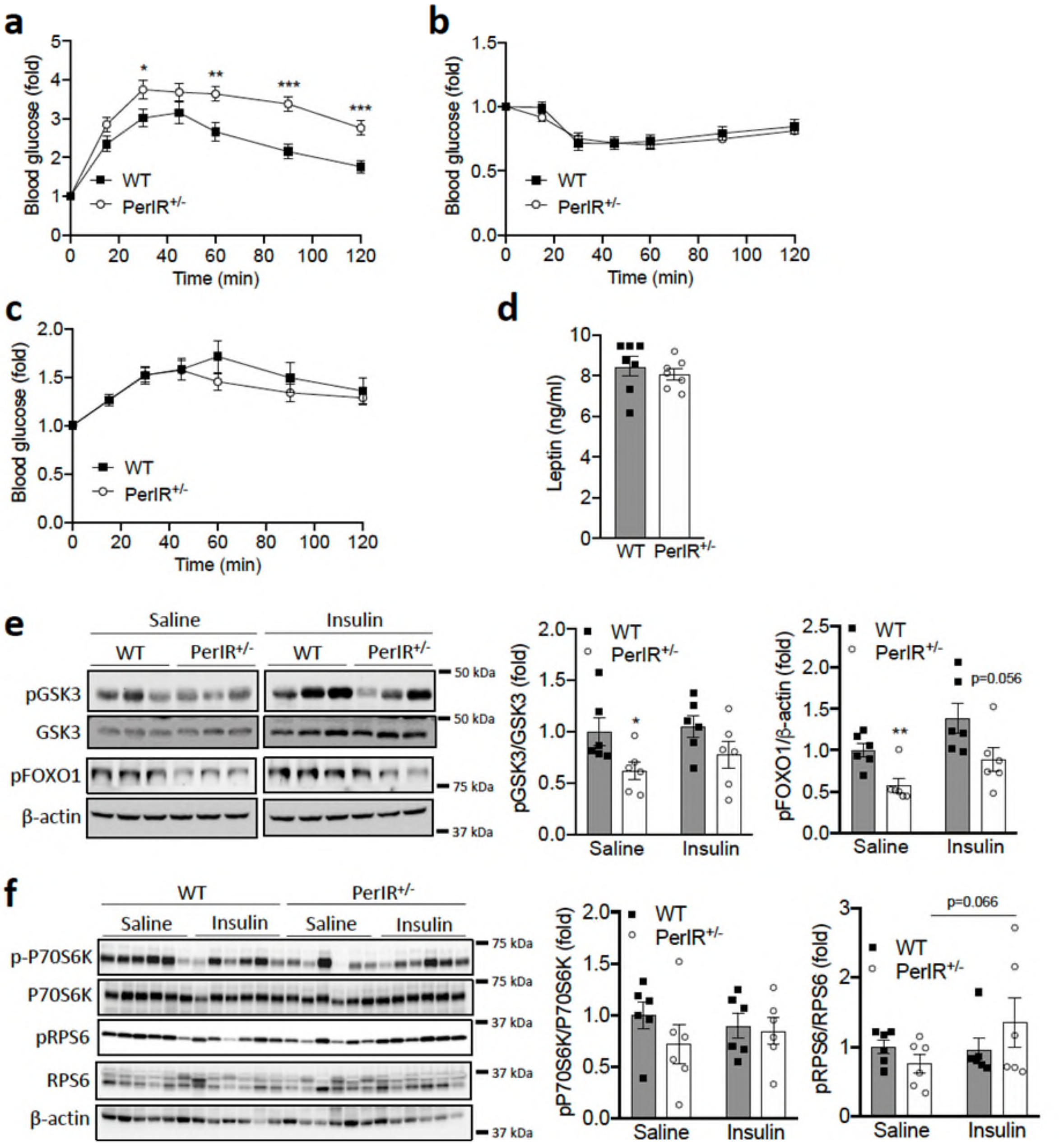

Supplementary Figure 2. Normalized glucose tolerance and insulin sensitivity, and downstream insulin and lipogenesis signaling in the livers of PerIRKO$^{+/-}$ mice fed a high fat diet. Ten days following tamoxifen (TX) treatment, male mice were fed a high fat diet (HFD) for 10 weeks and glucose homeostasis and insulin sensitivity was assessed via glucose (GTT) **(a)**, insulin (ITT) **(b)** and pyruvate (PTT) **(c)** tolerance test (normalized data from figure 2). Plasma leptin levels **(d)** and hepatic phosphorylation of GSK3 and FOXO1 **(e)**,

P70S6K and RPS6 **(f)** and in WT and PerIRKO$^{+/-}$ mice. Results are shown as means ± SE, with n's represented as individual data point's in figures. For tolerance tests n=11-12 per genotype. Significance was determined using two tailed student's t-test or ANOVA. *p < 0.05, **p < 0.01, ***p < 0.001 vs WT of same condition or timepoint.

Supplementary figure 3

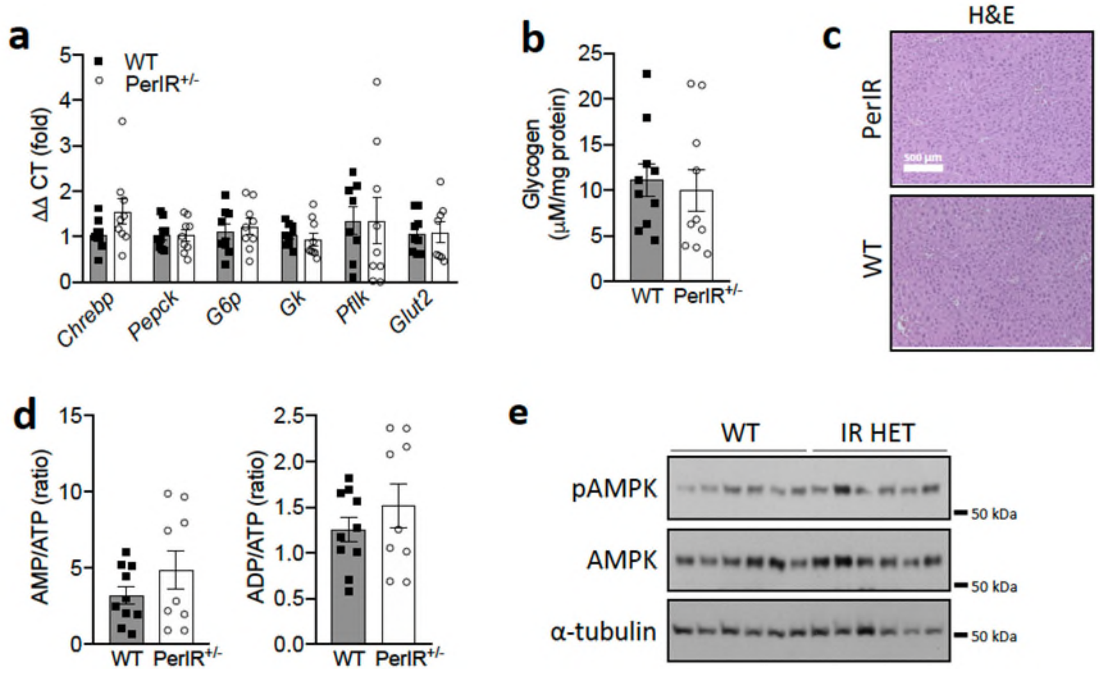

Supplementary Figure 3. Partial peripheral tissue IR disruption does not affect hepatic regulation in chow fed mice. Livers were collected from male WT and PerIRKO$^{+/-}$ mice 6 weeks following tamoxifen (TX) treatment, and glucogenogenic gene expression **(a)** and glycogen levels **(b)**, H&E staining **(c),** AMP, ATP, ADP levels **(d)** and AMPK phosphorylation **(e)** was determined. Results are shown as means ± SE, with n's represented as individual data point's in figures. For n=8-10 per group except for representative blots.
